# PD-L1 expression in colorectal cancer defines three subsets of tumor immune microenvironments

**DOI:** 10.18632/oncotarget.24196

**Published:** 2018-01-12

**Authors:** Anna Maria Valentini, Federica Di Pinto, Filomena Cariola, Vito Guerra, Gianluigi Giannelli, Maria Lucia Caruso, Michele Pirrelli

**Affiliations:** ^1^ Department of Pathology, National Institute of Gastroenterology “S. de Bellis”, Research Hospital, Castellana Grotte, Italy; ^2^ Medical Genetic Unit, National Institute of Gastroenterology “S. de Bellis”, Research Hospital, Castellana Grotte, Italy; ^3^ Department of Epidemiology, National Institute of Gastroenterology “S. de Bellis”, Research Hospital, Castellana Grotte, Italy; ^4^ National Institute of Gastroenterology “S. de Bellis”, Research Hospital, Castellana Grotte, Italy

**Keywords:** colorectal cancer, microsatellite instability, immunohistochemistry, PD-L1, PD-1

## Abstract

**Objectives:**

We investigated the PD-L1 expression in colorectal cancer (CRC) and in its microenvironment.

**Results:**

PD-L1 was expressed in neoplastic cells (NCs) and tumor-infiltrating immune cells (IICs). All samples PD-L1+ on NCs were also on IICs. Three types of cancers could be grouped: group A(NCs-/ IICs-); group B (NCs-/ IICs+); group C (NCs+/IICs+). To group A belong tumors characterized by poorly immunogenic competence, poor immune response but massive granulocyte infiltrate, justifying the absence of PD-L1 as an immunoinhibitor receptor. To Group B probably belong more immunogenic CRCs, justifying the strong IICs-mediated immune response, and up-regulation of PD-L1 expression only on IICs. To group C belong CRCs probably characterized by a large amount of tumor neoantigens resulting in a marked infiltration of lymphocytes and PD-L1 upregulation also in NCs.

**Materials and Methods:**

Sixty-three colorectal cancer specimens from a cohort of 61 patients were retrospectively reviewed. Thirty-seven MSS and 26 MSI-H CRCs enrolled in this study. Immunohistochemical staining to PD-L1 was performed by using MAb E1L3N.

**Conclusions:**

Our study calls attention to the importance to assess PD-L1 expression in tumor microenvironment also evaluating type and density of infiltrating immune cells to better stratify CRCs with different immunological patterns.

## INTRODUCTION

Colorectal carcinogenesis is driven by sequential genetic and epigenetic alteration of epithelial cells and is also influenced by tumor-host interaction [1–3]. A well-known example of this interaction is the strong histological reaction of tumor infiltrating lymphocytes (TILs) that globally constitute a robust prognostic parameter of favorable clinical outcome in colorectal cancer (CRC) patients, acting as the gatekeepers in preventing tumor dissemination [4]. However, tumors are rarely rejected spontaneously, because of their ability to create an immunosuppressive microenvironment through the activation of immune checkpoints such as PD-L1, CTLA-4, LAG-3, TIM-3 [5]. Programmed cell death-1 (PD-1) and PD-L1 (natural ligand of PD-1) pathway is one of the most studied immune checkpoint and it is crucial both in physiological conditions (for the maintenance of self-tolerance and to avoid autoimmune diseases) [6] and to evade antitumor immunity [7]. The specific binding between PD-1 and PD-L1 inhibits interleukin II production by T cells and increases the T lymphocyte apoptosis [8].

PD-1 is an immune-inhibitory receptor that is constitutively expressed by activated T lymphocytes and macrophages whereas other non-T lymphocytes, such as B cells and natural killer cells, express PD-1 only on induction [9, 10]. High levels of expression of PD-1 are induced on T lymphocytes as a result of persistent inflammatory stimuli that determine their exhaustion or anergy [11].

PD-L1 is constitutively expressed by T and B cells, macrophages and dendritic cells and is up-regulated by many inflammatory mediators and cytokines [12–14]. PD-L1 is also expressed on tumor cells in several cancer types and its expression implies a weakened host immune response and consequent poor prognosis in several malignancies as malignant melanoma, lung cancer (NSCLC), thymoma, bladder, ovarian, renal cell carcinomas (RCC) [15, 16], and GEP-NEN [17]. The prognostic role of PD-L1 in CRC is less clear, with some studies reporting conflicting results [18, 19].

Because immune checkpoints are one of the steps for immune escape and tumor development, inhibition of these molecules with monoclonal antibodies may restore host immune response and, consequently, stop tumor growth and even cause tumor regression. For this reason immunotherapy using immune checkpoint inhibitors is a rapidly growing modality for the treatment of some human cancer [5, 20–22]. One of the most studied predictive biomarkers for response to checkpoint blockade is the immunohistochemical expression of PD-L1. Studies have shown that inhibitors targeting PD-L1 or PD-1 protein can improve clinical outcomes in various types of cancers such as malignant melanoma, NSCLC, RCC, and bladder cancer. Although initial trials have suggested no role for immunotherapy in CRC [23], Le et al. [24] showed that 40% of patients with CRC-mismatch repair deficiency (dMMR), treated with Pembrolizumab (MAb anti-PD-1) responded to therapy vs 0% of patients with CRC-mismatch repair proficiency (pMMR) .

Although the majority of CRCs develops via a chromosomal instability pathway, approximately 12–15% have dMMR which is responsible for microsatellite instability (MSI) [25, 26]. This defect produce an increased mutational burden that leads to the creations of aberrant proteins, acting as neo-antigens eliciting an immune response by TILs [27, 28]. MSI CRC shows a T immune infiltration that is geographically associated with the upregulation of T cell checkpoints such as PD-L1. MSI CRCs are most frequently right-sided, poorly differentiated, mucinous, lymphocyte-rich neoplasias and show a more favorable prognosis than other types of CRCs.

Since it has been shown that not all MSI CRCs respond [24, 29], it is important to identify new targets that allow better selection of the eligible patients to immunotherapy.

The aim of this study is to investigate the immunohistochemical expression of PD-L1 in colorectal cancer cells and in its microenvironment and to correlate with microsatellite instability status and morphological and molecular characteristics.

## RESULTS

### PD-L1 immunohistochemical expression

A total of 63 CRC specimens were included in this study. The clinicopathological characteristics of patients are listed in Table 1.

**Table 1:** Clinicopathological and molecular characteristics of 63 CRCs

	*n* (%)
**Sex**	
Male	31 (50.8)^*^
Female	30 (49.2)^*^
**Age (M ± DS)**	58.87±14.84
**Tumor site**	
Right colon	31 (49.21)
Left colon	32 (50.79)
**Histological type**	
Adenocarcinoma	52 (82.54)
Medullary	11 (17.46)
**Tumor grade**	
G1+G2	33 (52.38)
G3	30 (47.62)
**Pattern of advancing border**	
Pushing	28 (44.44)
Infiltrating	35 (55.56)
**Tumor Budding**	
Absent	11 (17.46)
Present	52 (82.54)
**IICs**	
Absent/Mild	19 (30.16)
Moderated/Marked	44 (69.84)
**TANs**	
Sporadic	19 (30.16)
Massive	44 (69.84)
**pN status**	
N0	46 (73.02)
N1	17 (26.98)
**RAS status**	
Mutant	22 (34.92)
Wild-type	41 (65.08)
**BRAF status**	
Mutant	9 (14.28)
Wild-type	54 (85.72)
**MSI status**	
MSI	26 (41.27)
MSS	37 (58.73)

Abbreviations: TANs = Tumor Associated Neutrophils; IICs = Infiltrating Immune Cells.

^*^ = One male and one female have 2 synchronous tumors.

The male patient has both MSS tumors, the female has both MSI.

**Figure 1:**
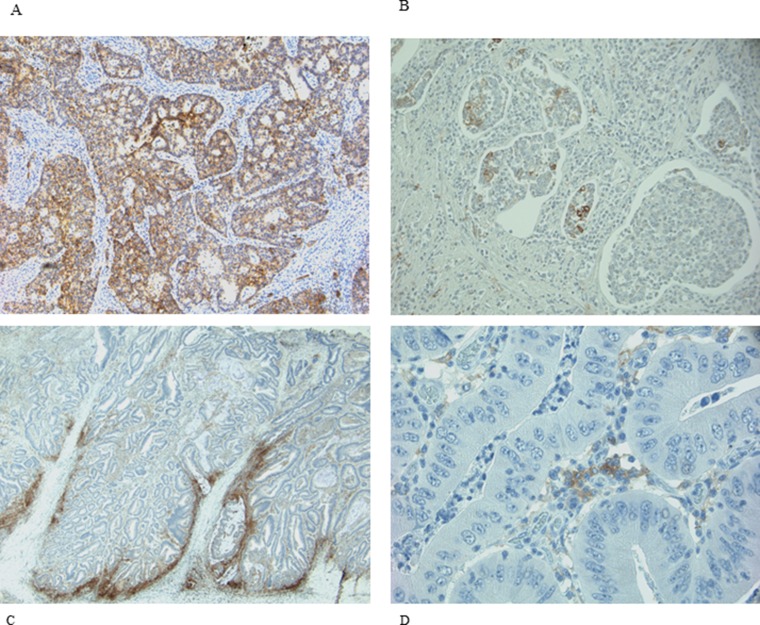
IHC expression of PD-L1 in NCs and IICs (**A** and **B)** representative diffuse and focal expression in NCs; (**C** and **D**) representative diffuse and focal expression in IICs.

PD-L1 staining was observed with different rate both in neoplastic cells (NCs) and in infiltrating immune cells (IICs). NCs were positive in 25% of samples, while IICs in 78% (Table 2). The normal colonic mucosa did not show any staining. A predominately membranous staining was observed in positive NCs cells. In these cells either a focal or a diffuse PD-L1 positive pattern, with the prevalence of the former was detected. When the pattern was focal, the positivity of NCs was prevalently localized along the tumor-stromal interface. Rarely scattered single or small nests of positive NCs dispersed in an otherwise negative tumor plate were observed. In IICs the membranous expression was more difficult to distinguish and a diffuse positive pattern was prevalent. Representative images of PD-L1 expression in NCs and IICs are shown in Figure 1.

**Table 2:** Clinicopathological and molecular characteristics of 63 CRCs with PD-L1 expression in NCs and in IICs

	PD-L1 expression					
	NCs			IICs		
	PD-L1 + *n =* 16 (25%)	PD-L1 - *n =* 47 (75%)	*P*-Value	PD-L1 + *n =* 49 (78%)	PD-L1 - *n =* 14 (22%)	*P*-Value
Sex			NS			NS
Male	9 (56.25)	25 (53.19)		25 (51.02)	9 (64.29)	
Female	7 (43.75)	22 (46.81)		24 (48.98)	5 (35, 71)	
**Age (M ± DS)**	66.25 ± 16.26	56.36 ± 13.62	**0.04**	59.90 ± 14.36	55.29 ± 16.48	NS
**Tumor site**			**0.004**			0.005
Right colon	13 (81.25)	18 (38.30)		29 (59.18)	2 (14.29)	
Left colon	3 (18.75)	29 (61.70)		20(40.82)	12 (85.71)	
**Histological type**			**< 0.001**			NS
Adenocarcinoma	6 (37.50)	46 (97.87)		38 (77.55)	14 (100)	
Medullary	10 (62.50)	1 (2.13)		11 (22.45)	0 (0)	
Tumor grade			**< 0.001**			NS
G1+G2	0 (0)	33 (70.21)		23 (46.94)	10 (71.43)	
G3	16 (100)	14 (29.79)		26 (53.06)	4 (28.57)	
**Pattern of advancing border**			NS			NS
Pushing	7 (43.75)	21 (44.68)		22 (44.90)	6 (42.86)	
Infiltrating	9 (56.25)	26 (55.32)		27 (55.10)	8 (57.14)	
**Tumor Budding**			NS			NS
Absent	1 (6.25)	10 (21.28)		8 (57.14)	3 (21.43)	
Present	15 (93.75)	37 (78.72)		41 (83.67)	11 (78.57)	
**IICs**			NS			**<0.001**
Absent/Mild	3 (18.75)	16 (34.04)		7 (14.28)	12 (85.71)	
Moderated/Marked	13 (81.25)	31 (65.96)		42 (85.72)	2 (14.29)	
**TANs**			**0.003**			NS
Sporadic	10 (62.50)	9 (19.15)		14 (28.57)	5 (35.71)	
Massive	6 (37.50)	38 (80.85)		35 (71.43)	9 (64.29)	
**pN status**			NS			NS
N0	9 (56.25)	37 (78.72)		37 (75.51)	9 (64.29)	
N1	7 (43.75)	10(21.28)		12 (24.49)	5 (35.71)	
**RAS status**			NS			NS
Mutant	5 (31.25)	17 (36.17)		17 (34.69)	5 (37.51)	
Wild-type	11 (68.75)	30 (63.83)		32 (65.31)	9 (64.29)	
**BRAF status**			**0.001**			NS
Mutant	7 (43.75)	2 (4.26)		9 (18.37)	0 (0)	
Wild-type	9 (56.25)	45 (95.74)		40 (81.63)	14 (100)	
**MSI status**			**0.003**			**0.03**
MSI	12 (75.00)	14 (29.79)		24 (48.98)	2 (14.29)	
MSS	4 (25.00)	33 (70.21)		25 (51.02)	12 (85.71)	

Abbreviations: NCs = Neoplastic Cells; TANs = Tumor Associated Neutrophils; IICs = Infiltrating Immune Cells.

NS = not significant.

### PD-L1 in NCs

The association between PD-L1 expression in NCs and clinicopathological and molecular parameters is shown in Table 2. PD-L1 expression was significantly associated with older age (*p* = 0.04), right sided location (*p* = 0.004), medullary histology (*p* < 0.001), G3 tumor grade (*p* < 0.001), sporadic tumor associated neutrophils (TANs) (*p* = 0.003), *BRAF* mutation (*p* = 0.001), and MSI (*p* = 0.003). On the contrary, the other features such as sex, pattern of advancing border, tumor budding, IICs density, pN status, and *RAS* status were not associated with PD-L1 expression in tumor cells. By multivariate analysis, after adjusting for sex and age, right sided location (OR 6.25; 95% CI 1.44–27.17; *p* = 0.01), medullary histology (OR 62.81; 95% CI 6.61–596.77; *p* < 0.001), sporadic TANs (OR 6.41; 95% CI 1.70–24.13; *p* = 0.006), N1 status (OR 5.33; 95% CI 1.20–23.66; *p* = 0.03), *BRAF* mutation (OR 18.41; 95% CI 2.15–157.93; *p* = 0.008) and MSI status (OR 10.79; 95% CI 2.37–49.12; *p* = 0.002) were significantly associated with PD-L1 expression.

### PD-L1 in IICs

The association between PD-L1 expression in IICs and clinicopathological and molecular parameters is shown in Table 2. PD-L1 expression in IICs was significantly associated with right sided location (*p* = 0.005), moderate/marked IICs density (*p* < 0.001), and MSI (*p* = 0.03). Instead the other features (sex, age, histological type, tumor grade, pattern of advancing border, tumor budding, TANs, pN status, *RAS* and *BRAF* status ) were not associated with PD-L1 expression in IICs.

By multivariate analysis, after adjusting for sex and age, right sided location (OR 7.94; 95% CI 1.51–41.70; *p* = 0.01) and MSI status (OR 5.48; 95% CI 1.06–28.21; *p* = 0.04) were significantly associated with PD-L1 expression.

### Pattern of PD-L1 expression in tumor microenvironment

All specimens PD-L1 positive in NCs showed PD-L1 expression also in IICs. There were no specimens positive only in NCs. Following these findings three groups can be assembled: group A (14 samples negative both in NCs and in IICs); group B (33 samples positive only in IICs); group C (16 samples positive both in NCs and IICs). The comparison among groups is shown in Table 3. Samples of group A compared with those of group B are prevalently localized on the left colon (*p* = 0.01). Samples of group C compared with those of group A are prevalently localized on the right colon (*p* < 0.0001), are associated with medullary histological type (*p* < 0.0001), G3 tumor grade (*p* < 0.0001), *BRAF* mutation (*p* = 0.001) and MSI status (*p* < 0.0001). Samples of group B compared with those of group C are prevalently localized on the left colon (*p* = 0.02), are associated with adenocarcinoma histological type (*p* < 0.0001), G1/G2 tumor grade (*p* < 0.0001), massive TANs (*p* = 0.0005), N0 status (*p* = 0.05), *BRAF* wild-type (*p* = 0.007) and microsatellite stability status (MSS) (*p* = 0.008).

**Table 3:** Comparison between groups A , B, C

	A NCs-/IICs- *n =* 14 (23%)	B NCs-/IICs+ *n =* 33 (52%)	C NCs+/IICs+ *n =* 16 (25%)	*P*-Value	Comparison		
					A vs B	C vs A	B vs C
**Sex**				NS			
Males	9 (64.29)	16 (48.48)	9 (56.25)		NS	NS	NS
**Age (M ± DS)**	55.28±16.47	56.82±12.47	66.25±16.26	NS	NS	NS	NS
**Tumor site**				**0.01**			
Right colon	2 (14.29)	16 (48.48)	13 (81.25)		**0.01**	**<0.0001**	**0.02**
**Histological type**				**<0.001**			
Medullary	0 (0.00)	1 (3.03)	10 (62.50)		NS	**<0.0001**	**<0.0001**
Tumor grade				**<0.001**			
G3	4 (28.57)	10 (30.30)	16 (100.00)		NS	**<0.0001**	**<0.0001**
**Pattern of advancing border**				NS			
Infiltrating	8 (57.14)	18 (54.55)	9 (56.25)		NS	NS	NS
Tumor Budding				NS			
Present	11 (78.57)	26 (78.79)	15 (93.75)		NS	NS	NS
**IICs**				**<0.001**			
Absent/Mild	12 (85.71)	4 (12.12)	3 (18.75)		**<0.0001**	**<0.001**	NS
**TANs**				**0.001**			
Sporadic	5 (35.71)	4 (12.12)	10 (62.50)		NS	NS	**0.0005**
**pN status**				NS			
N1	5 (35.71)	5 (15.15)	7 (43.75)		NS	NS	**0.05**
**RAS status**				NS			
Mutant	5 (35.71)	12 (36.36)	5 (31.25)		NS	NS	NS
**BRAF status**				**0.001**			
Mutant	0 (0.00)	2 (6.06)	7 (43.75)		NS	**0.001**	**0.007**
**MSI status**				**0.003**			
MSI	2 (14.29)	12 (36.36)	12 (75.00)		NS	**0.0001**	**0.008**

Abbreviations: NCs = Neoplastic Cells; TAN s= Tumor Associated Neutrophils; IICs = Infiltrating Immune Cells.

NS = not significant.

## DISCUSSION

PD-1/PD-L1 pathway is a significant mechanism of immune suppression within tumor microenvironment. MAbs to PD-1 and PD-L1 could block this pathway and reverse T cell exhaustion inducing tumor regression. In the treatment of malignancies including melanoma, NSCLC, renal cell carcinoma, bladder cancer these MAbs have an overall response rates ranging from 16% to 100%[30, 31]. Although initial trials have suggested no role for immunotherapy in patients with CRC, more recently it has been demonstrated that a specific subset of patients did benefit [4, 29].

Le et al. [24] in a phase II clinical trial, after the administration of Pembrolizumab (an anti-PD-L1 Mab), showed a partial objective response rate of 40% and 0% for dMMR and pMMR CRC patients, respectively. The phase II trial by Overman et al. [29] is, to date, the largest immunotherapy trial in CRC and highlighted a partial response to Nivolumab alone or in association with Ipilimumab in 31% of MSI patients versus 10% of MSS patients.

Despite these results being encouraging, it is clear that most MSI CRCs do not respond to immunotherapy and this represents the rational for testing other markers able to predict cancer population who be eligible for personalized immune approaches.

There is an apparent paradox: both MSI CRC and medullary carcinoma which have many overlapping clinical and morphological features, are generally characterized by better prognosis [32]. It is believed that this is associated with the rich lymphocyte infiltration which would give a robust immune response against the tumor. On the other hand, the prognostic unfavorable significance of PD-L1, often expressed by both of these tumor types, appears to be in contrast to the well-known favorable prognosis of MSI CRC and medullary carcinoma. However, not all MSI CRCs and medullary carcinomas have better survival [33]. Furthermore, the analysis of different types of tumors showed that PD-L1 status may correlate with either good or bad prognosis, or have no influence [34–38]. Therefore, if the expression of PD-L1 on NCs membrane could be the trick to escape an efficient immune-mediated antitumor response, then PD-L1 positivity in NCs of MSI CRC and/or medullary histological type, might be the predictive marker of that subset of samples with worse prognosis.

PD-L1 immunohistochemical expression in NSCLC and melanoma is used as marker to select patients who may be successful in the treatment with Pembrolizumab and Nivolumab [30, 31].

Assessing PD-L1 expression is complicated by many challenges: different immunohistochemical assays (i.e. different primary antibodies and assays conditions) and different PD-L1 evaluation methods (i.e. different scoring methods and PD-L1 positivity cut-offs) complicate the comparative evaluation between clinical studies [11, 18, 27, 39–44].

We used MAb E1L3N to PD-L1 immunohistochemical detection and cut-off of 5% positive cells. Most studies on CRC evaluated the expression of PD-L1 using a 1% or 5% cut-off value [39–44]. We used the latter value for the objective difficulties in the detection of very rare positive neoplastic cells and to avoid overestimating of PD-L1 expression. In addition, like few other studies [39–41], we evaluated PD-L1 not only in neoplastic cells but also in immune cells.

**Figure 2:**
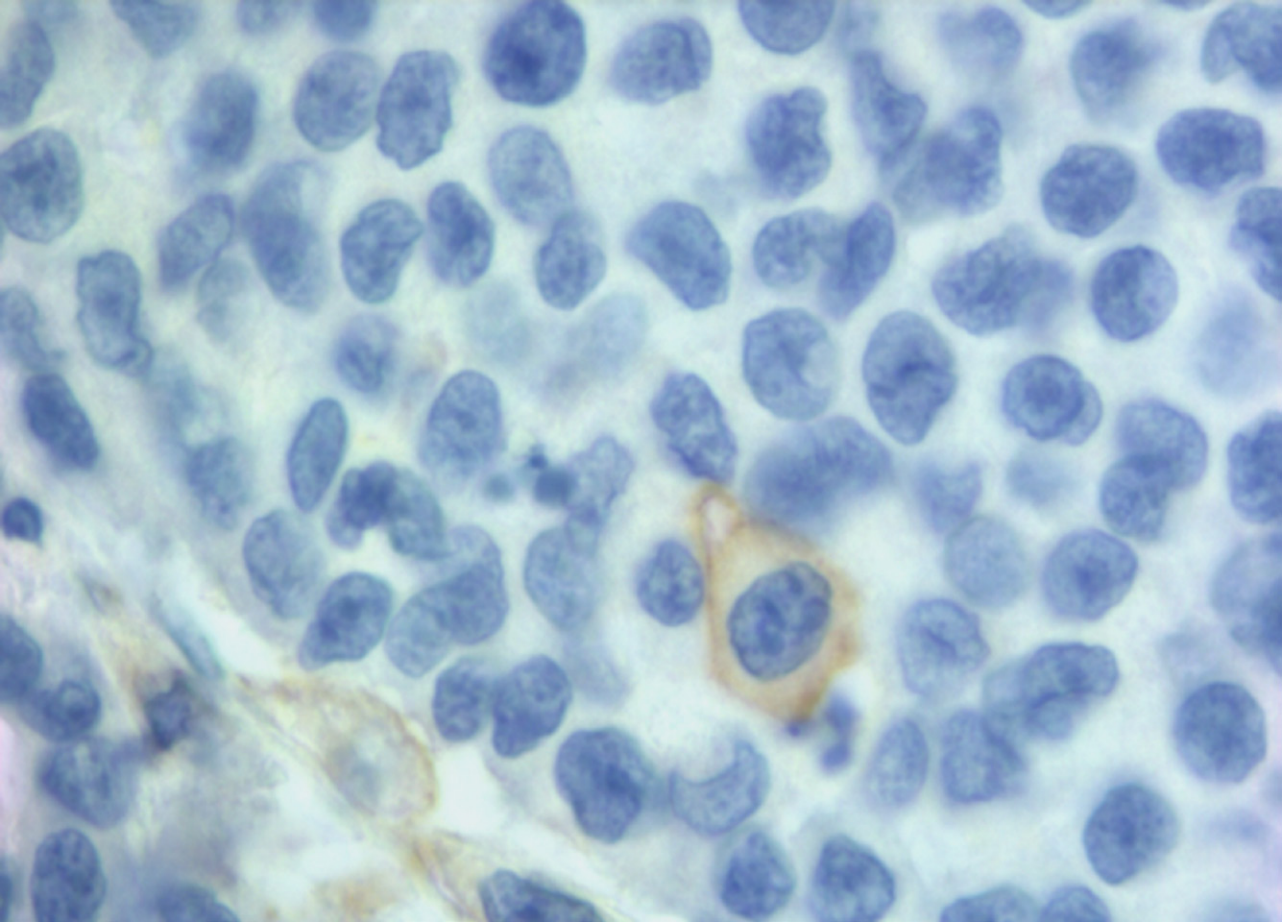
A clear membranous IHC expression of PD-L1 in a single neoplastic cell

We found 25% of cases with PD-L1 positive in NCs and 78% in IICs. Our positivity rate is higher than that described in other studies using the same MAb E1L3N and the same 5% cut-off [11, 39, 42, 43]. Our results can be due to several factors. We carried out the immunohistochemical detection on whole section of tissue versus most of the studies carried on tissue microarrays (TMA). The limited amount of TMA tissue could underestimate focal expression of PD-L1. Indeed in most our samples there was a focal PD-L1 positivity in NCs (Figure 1). In addition, unlike Kim et al. [39], we evaluated the membranous positive staining regardless intensity, including the cases with weak but clear immunohistochemical staining. We think that the peculiar membrane stain (Figure 2) compared to the absence of PD-L1 expression is sufficient to categorize as positive also specimens with weak intensity of staining. Furthermore, to enrolling our cohort, in addition to MSI we selected 11 medullary carcinoma samples of which 10 resulted positive to PD-L1, helping to increase the rate of positivity.

Thus, in agreement with the literature [11, 39, 42, 43, 45], we found that the expression of PD-L1 on NCs is significantly associated with older age, right sided location, medullary histology, G3 tumor grade, *BRAF* mutation, and MSI status (Table 2). In addition we also found an association between PD-L1 expression in NCs with the sporadic TANs.

On the contrary, PD-L1 expression in IICs is significantly associated with right sided location (*p* = 0.005), moderate/marked IICs (*p* < 0.001), and MSI (*p* = 0.03) (Table 2).

Our results have clearly shown that there is no positivity of PD-L1 in NCs without positivity in IICs. These data allows to outline three types of tumors: tumor PD-L1 negative both in NCs and in IICs (group A), tumors PD-L1 positive only in IICs (group B), tumors PD-L1 positive both in NCs and IICs (group C). The association between the three groups and the variables in observation are shown in Table 3.

Many cells with immune functions populate the tumor microenvironment: T cells, B cells, NK cells, macrophages, neutrophils, dendritic cells. This heterogeneous population, together with stromal cells such as fibroblasts and endothelial cells, contribute to forming an activated immune system against the cancer. The crosstalk between these different cellular populations exploits cytokines and other molecules that interact with other cells in the microenvironment. Unfortunately the complex of mechanisms that regulate the antitumor response of the immune system is yet to be completely elucidated.

In CRC, especially MSI ones, it is believed that the neo-epitopes burden produced by a large number of mutation induces a T-cell mediated immune-response with three possible sequential scenarios. In a first phase (elimination phase), the stimulated immune system produced many costimulating cytokines (TNFα, IL1, IFNα, TLR, ICAM1, IL2, IL12, IFNγ,) and less inhibitors (IL10, IL4, IL13, PD1/PD-L1, prostaglandins, LAG-3, TGFβ) resulting in efficient tumor cell killing. Subsequently, an equilibrium phase takes over, in which cancer cells activate biochemical pathways inducing a dynamic state of immune tolerance. The third phase (escape phase) begins when cancer cells become capable of binding and activating the co-inhibitory molecules on the T lymphocytes (CTLA-4, PD-1, LAG-3, TIM-3), and secreting soluble immunosuppressive or inducing the secretion of mediators such as IDO, TGFβ, IL10. The final result is the blockade of the immune system and the cancer survival [2].

In addition to T-mediated mechanism, another line of defense against cancers are neutrophil granulocytes, the most abundant leukocytes. Recent studies have shown the independent unfavorable prognostic role in several humans tumor [46–50] including CRC [51].

Shaul et al. [52], in a murine model, have shown that TANs has two different patterns of expression against tumor. The first pattern (N1) has an antitumorigenic phenotype, the second (N2) has a protumorigenic phenotype, working with opposite regulatory balancing, probably TGFβ and IFNβ mediated. High levels of TGFβ and low levels of IFNβ would elicit the protumorigenic phenotype of TANs. In addition, it has been shown that TANs isolated in early stage tumours would be more cytotoxic than those isolated in advanced tumors. So the transition from N1 to N2 phenotype would occur during tumor progression. In N1 phenotype TANs express high levels of proinflammatory cytokines and chemoattractive substances for macrophages and T cells. On the contrary, TANs with N2 phenotype secrete CCL-17 with consequent recruitment of CD4 Treg displaying protumour activity. Indeed depletion of neutrophils reduces the CD4 Treg chemoattractive effect [53].

Therefore, a delicate and still not fully known crosstalk mechanism seem to play a fundamental role in determining the density and function of immune population involved in the tumor microenvironment.

The data of this small retrospective study on a CRC cohort with and without microsatellite instability clearly demonstrate that the type and density of inflammatory cells populating the tumor microenvironment together with PD-L1 expression identify three different tumor groups (A, B, C).

Group A, maybe due to neo-antigen scarcity, shows a poor IICs-mediated immune response and a strong granulocytes-mediated immune response. It would then be formed by very little immunogenic CRCs, so that the lymphocyte response is absent/mild and on the contrary it is massive the granulocytic one. In fact to the group A belongs prevalent MSS CRCs and not PD-L1 expressing (both in NCs and IICs) tumors.

Group B, maybe due to the presence of more neo-antigens, has a strong IICs-mediated immune response, with or without granulocyte participating and the positivity of IICs to PD-L1 is probably linked to the perfect and vigorous functioning of the immune response that elicits self-control mechanisms. To this group belong a prevalence of MSS CRCs. In addition, Housseau et al. [54] suggest that in MSS CRCs the nature more than the number of intratumor mutations may drive immunogenicity. This could explain the immunogenic features of some MSS CRC belonging to B group.

Group C, often shows a moderated/marked component of IICs with sporadic TANs. In this group there is probably a remarkable neo-antigens production with an equally remarkable T-cell immuno-mediated response counterbalanced by checkpoint inhibitor signaling, e.g. PD-L1, on both IICs and NCs, that would guarantee tumor tolerance and favor tumor progression. Indeed in group C there is an high percentage of MSI and medullary carcinomas, probably the subset of these tumors with unfavorable prognosis.

If this theoretical setting is true, you might suggest that therapy with immune checkpoint inhibitors is useless in group A because another type of response works, while it might be strongly recommended in group C where the immunological response could be exhausted.

Group B is less clearly outlined. It is generally conspicuous both the IICs and the TANs. Although the biologic significance of this distinct pattern of PD-L1 expression is currently not clear, it likely reflects the combined effect of innate and adaptive cellular and soluble factors that shape the tumor microenvironment [44]. In addition, the presence of a significant TANs infiltrate indicates that the response of the entire immune system is likely to be more complex and involves several actors with different roles.

In conclusion our study shows that: it is important the comprehensive assessment of PD-L1 expression both in NCs and IICs; PD-L1 on NCs may have heterogeneous and focal expression; in agreement with other published data in CRCs the PD-L1 expression on NCs associates with right sided tumors and high grade, medullary histological type, MSI status, marked IICs; the granulocyte infiltration also contributes to separating CRCs with different immune microenvironments; the combination of density and type of inflammatory cells together with the expression of PD-L1 on NCs and IICs defines three tumor groups variously associated to MSI status, medullary histology and other clinicopathological features; theoretically the group A (high TANs, low IICs, PD-L1 negative) would not benefit from anti-PD-L1 therapy otherwise the third group (low TANs, high IICs, both IICs and NCs PD-L1 expressing) would be the ideal candidate for this type of therapy; further studies are needed to validate these hypotheses and to check within the group B (high TANs and IICs, PD-L1 expressing exclusively on IICs ) other predictive features able to select candidate patients for the blockade immunotherapy.

## MATERIALS AND METHODS

### Patients and tumor characteristics

This study encompasses 63 formalin-fixed paraffin-embedded colorectal carcinoma specimens from 61 patients (31 males and 30 females) underwent surgical resection (between the years of 2010 and 2016) at our National Institute of Gastroenterology.

For selection, we have reviewed written pathology reports searching for keywords “adeno”, and “medullary” and in addition, within these histological types, we have selected those carcinomas with known MSS or MSI status. All the tumors had been surgically resected in a single surgery unit of our Institute and underwent pathological and molecular testing in our molecular pathology laboratory.

Therefore, we collected 37 MSS (33 adenocarcinomas and 4 medullary), 26 MSI-H (19 adenocarcinomas and 7 medullary) CRCs. All MSI were sporadic CRCs.

**Figure 3:**
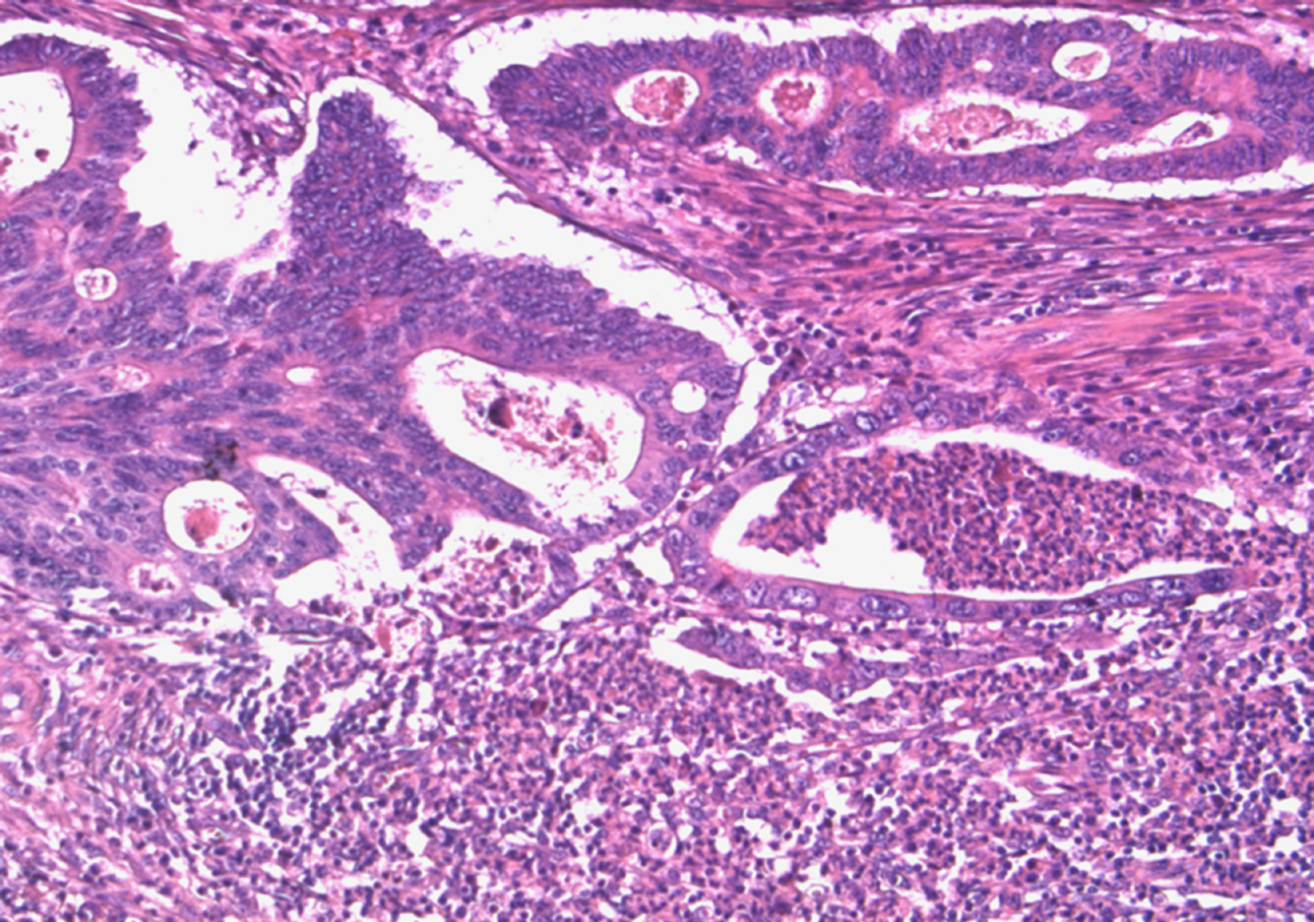
Massive TANs infiltration at tumoral edge with abscessual features

The same paraffin block on which molecular assays had been performed was chosen. Hematoxylin and eosin-stained sections of all formalin-fixed paraffin-embedded blocks were reviewed by a pathologist (MP) to confirm the presence of adequate tumor tissue and to evaluate tumor grade, pN status, pattern of advancing border, tumor budding, TANs, and IICs encompassing intra and peritumoral lymphocytes, macrophages, plasma cells. The IICs were evaluated as absent/mild when few cells were highlighted within the tumor and/or at the tumor-stroma interface, moderate/marked when the infiltrate was of greater density with the tendency to flow into plagues and infiltrate neoplastic epithelium. We evaluated TANs at the tumor-stroma interface and graduated as sporadic (Supplementary Figure 1) when only a few and spread granulocytes were present and massive when they were much more abundant and confluent into stromal or intraglandular abscesses (Figure 3). The study was approved by the institutional review board.

### Immunohistochemistry

Immunohistochemistry (IHC) analysis was carried out in sections (4 micron thick) mounted on positively charged slides and dewaxed in an oven for 20’ at 60°C followed by two serial xylene dippings. Sections were then rehydrated in grades alcohols and incubated in 3% hydrogen peroxide for 10’ to block endogenous peroxidase activity. Sections were retrieved in EDTA buffer (pH 8) at 98°C for 30′. For PD-L1 IHC, has been used a monoclonal antibody against cytoplasmic domain of PD-L1 (clone E1L3N, dilution 1:800; Cell Signaling Technology, Danvers, MA, USA), on an automated autostainer (Dako, Glostrup, Denmark). The dilution factor of this antibody and target retrieval buffer were optimized by using tonsil and placental tissues as positive control.

Dako Real ENVISION (Dako, Glostrup, Denmark) was used as visualization reagent and the 3,3’-DAB as chromogen, according to the manufacturer´s instructions.

Human placenta was included as positive control on each staining run. Negative rabbit IgG controls were used and did not show any staining.

PD-L1 immunoreactivity was evaluated separately for NCs and IICs. PD-L1 positivity was defined as PD-L1 expression on ≥ 5% of membranous positive cell staining of any intensity. Cytoplasmic staining was not considered in this study. The immunostains were independently evaluated by two pathologists (MLC, MP). For discordant cases, the results were confirmed by discussion of the two pathologists.

CD3 (Polyclonal antibody, dilution 1:200; Dako, Glostrup, Denmark) and CD68 (clone KP1, dilution 1:1000; Dako, Glostrup, Denmark) immunostains were performed on each specimen by using the same protocol of PD-L1, except antigen retrieval that was carried out in 10mM Citrate buffer (pH 6). The CD3 and the CD68 stains were used to better interpret the topographical distribution and the composition of PD-L1 expressing immune infiltrate (Supplementary Figure 2).

### KRAS, NRAS, and BRAF Mutation analysis

The protocols were performed according to the manufacturer’s instructions. Two sections (10 micron thick) of FFPE tissue were used for DNA extraction using the QIAamp DNA FFPE Tissue Kit (Qiagen, Hilden, Germany). As a first step we evaluated, by pyrosequencing (Pyromark Q24, Qiagen, Hilden, Germany), the status of *KRAS* codons 12, 13 of all specimens enrolled in this study. Later, on 12/13 wild type samples, it was performed *RAS* extension (*KRAS* codons 59, 61, 117, 146 and *NRAS* codons 12, 13, 59, 61,117, 146) and *BRAF* (V600E) analysis [55]. Briefly, 50 ng of genomic DNA combined with codon-specific primers were used for the initial 25 µL PCR reaction volume. After PCR, 10 µL of the amplicons were immobilized on streptavidin-coated beads and denatured to produce single-stranded products. Single-stranded DNA was prepared and the corresponding sequencing primers were allowed to anneal to the DNA. Sequence analysis was performed using PyroMark Q24 software in the AQ (allele quantification) analysis mode. The quality thresholds for the mutational analysis were a required peak height of 30 relative units for “passed” quality and 10 relative units for “check” quality. A negative control (without DNA) and a wild type control were run with each series of samples.

Samples with a reported potential low-level mutation were considered positive for the mutation, if confirmed by re-running in duplicate with wild type DNA control.

### MSI testing

MSI testing was performed on paraffin-embedded tumor tissue using a PCR-based assay with five National Cancer Institute recommended markers (BAT25, BAT 26, D5S346, D17S250, and D2S123) [56, 57].

MSI testing required DNA from both normal and tumor tissue for each patient. PCR amplification was performed in a total reaction volume of 20 µl, containing 100 µM dNTPs, 0.5 µM each primer, 0.25 U of Taq Polymerase Platinum, 1.5 mM MgCl_2_ and 200 ng DNA. PCR cycling conditions were: denaturation at 95° for 5 minutes, followed by 30 cycles of 95°C for 30 seconds, 55°C for 30 seconds, 72°C for 30 seconds, and a final elongation at 72°C for 5 minutes. The PCR products were controlled on 1.5% agarose gel and were separated on a vertical denaturing 6M Urea polyacrilamide gel (acrylamide/bis-acrylamide 19:1). Polyacrilamide gel was stained with Silver Nitrate. On the basis of the MSI status, CRCs can be classified into three groups: MSI-Low if only one of the five microsatellite markers shows instability, MSI-High if two or more of the five microsatellite markers show instability and MSS if none of the markers shows instability [56, 57].

### Statistical analysis

Means and standard deviations (M ± SD) for continuous variables, and relative frequency (%) for categorical variables, were used as indices of centrality and dispersion of the distribution.

Chi-square test or Fisher exact test, depending on the number of observations, for categorical variables, Kruskal-Wallis rank test and Wilcoxon rank-sum test (Mann-Whitney test) for continuous variables were used to test differences among the groups. In the logistic regression models the absence of PD-L1 expression was considered as a reference category.

All models were adjusted for gender and age. Results were presented as Odds-Ratio (OR) and with 95% Confidence Intervals (C.I.). The Odds-Ratio represents the risk for one-unit variation of the predictor variable. When testing the hypothesis of significant association, *p*-value was < 0.05, two tailed for all analysis. All statistical computations used STATA 10.0 Statistical Software, (StataCorp), College Station, TX, USA.

## SUPPLEMENTARY MATERIALS FIGURES


