# Supplementary material for: PD-L1 expression in colorectal cancer defines three subsets of tumor immune microenvironments
Source: Oncotarget. 2018 Jan 12;9(9):8584–96. doi: 10.18632/oncotarget.24196 (PMC5823560; doi:10.18632/oncotarget.24196)
Supplement: Supplementary file 1 [file oncotarget-09-8584-s001.pdf]

PD-L1 expression in colorectal cancer defines three subsets of tumor immune microenvironments

SUPPLEMENTARY MATERIALS

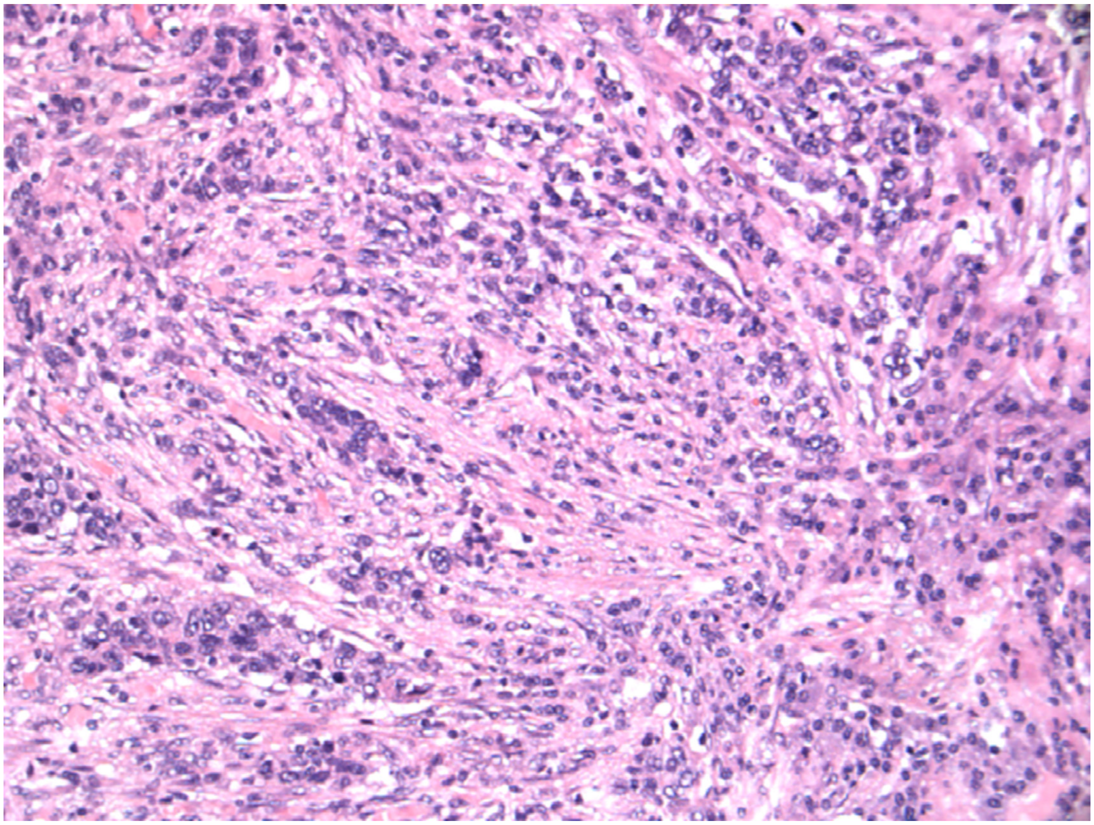

Supplementary Figure 1: Sporadic TANs infiltration at tumoral edge.

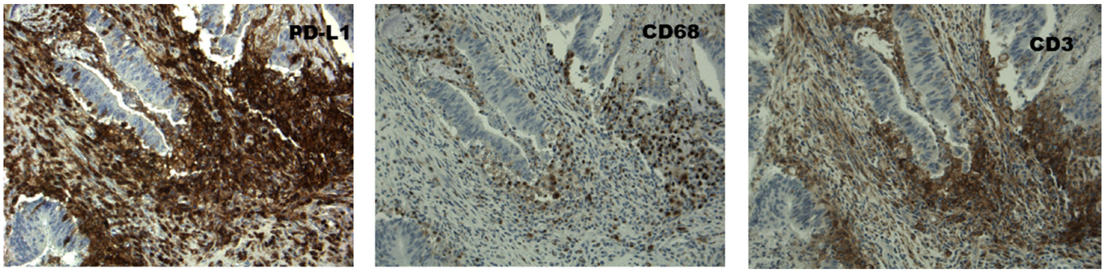

Supplementary Figure 2: Immunohistochemical stainings of PD-L1, CD68, and CD3 (same field).
